# Medizinische Priorisierung in Pandemien und der ethische Diskriminierungsbegriff

**DOI:** 10.1007/s00481-023-00765-7

**Published:** 2023-04-27

**Authors:** Annette Dufner

**Affiliations:** grid.7491.b 0000 0001 0944 9128 Abt. Philosophie und FB Medical Humanities, Universität Bielefeld, Postfach 10 01 31, 33501 Bielefeld, Deutschland

**Keywords:** Priorisierung, Diskriminierung, Triage, Behinderungen, Gebrechlichkeit, Priority setting, Discrimination, Triage, Disability, Frailty

## Abstract

Im Falle von Pandemien können die Kapazitäten auf den Intensivstationen bekanntlich knapp werden. In Deutschland hat dieser Umstand zu einem Urteil des Bundesverfassungsgerichts geführt, in dem vom Gesetzgeber verlangt wurde, Menschen mit Behinderungen für solche Fälle besser vor Diskriminierung zu schützen. Aus ethischer Sicht hängt die Frage nach den Diskriminierungspotentialen von Priorisierungskriterien stark davon ab, worin genau das Übel einer Diskriminierung verortet werden muss – eine Frage, zu der es in der Ethik mehrere konkurrierende Theoriegruppen gibt. In diesem Aufsatz soll gezeigt werden, dass diese Theorien darüber hinaus jeweils einer Ergänzung bedürfen, um dem Phänomen der indirekten Diskriminierung gerecht werden zu können. Wie hier am Beispiel einiger konkreter Triage-Kriterien gezeigt werden soll, ist ein Ansatz mittlerer Breite besonders geeignet, um die Aufmerksamkeit auf den Kern der aktuellen Probleme zu lenken. Dazu zählt insbesondere das Ausmaß, in dem soziale Gepflogenheiten gegenüber Menschen mit bereits zuvor bestehenden konkreten Schwierigkeiten generell Auswirkungen auf die Struktur ihrer sozialen Interaktionen haben.

## Abstract (en)

**Definition of the problem:**

As we have all learned in recent years, a pandemic can produce shortages in intensive care units. In our jurisdiction, this has led to a ruling by the federal constitutional court, according to which the lawmaker has to provide better protection for persons with disabilities in the event of medical priority setting.

**Arguments:**

From an ethical perspective, this task requires a choice among various competing accounts on what exactly it is that makes a case of discrimination morally problematic. In addition, these accounts require amendments in order to encompass instances of indirect discrimination.

**Conclusion:**

As this article demonstrates by appeal to a number of concrete triage criteria, a moderate account of discrimination does the best job of focusing attention on the core of the current issues. Among these issues are the extent to which perceptions of persons with specific pre-existing challenges generally have an impact on the structure of their social interactions.

Zwei Jahre nach Beginn der Covid-19-Pandemie sind Expertinnen und Experten aus Medizin, Recht, Ethik und Politik nach wie vor mit der Aufarbeitung drohender Knappheitssituationen auf den Intensivstationen befasst. Prioritätensetzungen für möglicherweise lebensrettende medizinische Zuteilungsentscheidungen dürfen nach gängiger Auffassung keine Diskriminierungen gegen Menschen mit Behinderungen mit sich bringen. Auch das Bundesverfassungsgericht schloss sich dieser Auffassung im Dezember 2021 an. Der Gesetzgeber sei hier, so heißt es von Seiten des Gerichts, seinen Schutzpflichten gegenüber Menschen mit Beeinträchtigungen nicht nachkommen^1^ und müsse dies nachholen^2^ – was mittlerweile in Form einer Ergänzung des Infektionsschutzgesetzes auch geschehen ist. Der neue § 5c verbietet nun explizit Zuteilungsentscheidungen auf Grundlage „einer Behinderung, des Grades der Gebrechlichkeit, des Alters, der ethnischen Herkunft, der Religion oder Weltanschauung, des Geschlechts oder der sexuellen Orientierung“. Grundlage der Entscheidung solle jedoch weiterhin die „kurzfristige Überlebenswahrscheinlichkeit“ der Betroffenen sein.

Das Verfassungsgerichtsurteil und die listenartige Herangehensweise an das Problem im Infektionsschutzgesetz weisen zahlreiche interessante Aspekte auf und lenken den Blick unter anderem auf die Frage, was eigentlich genau unter einer Diskriminierung zu verstehen ist. Eine Auffassung, der sich das Verfassungsgericht dabei *nicht* angeschlossen hat – und die sich auch bei der Ergänzung des Infektionsschutzgesetzes nicht durchsetzen konnte –, besteht darin, anzunehmen, jegliche erfolgsorientierte Vergabe von Intensivbetten in einer Pandemie sei unweigerlich problematisch^3^ und mit Diskriminierungen von Menschen mit bestehenden Beeinträchtigungen verbunden. Im Verfassungsgerichtsurteil wurde stattdessen lediglich auf die Gefahr einer stereotypisierten Einschätzung der Erfolgsaussichten von Menschen mit Beeinträchtigungen abgestellt.^4^ Das Gericht trägt damit dem Umstand Rechnung, dass die in zahlreichen Ländern entstandenen Empfehlungen der medizinischen und medizinethischen Fachgesellschaften^5^ eine an den Erfolgsaussichten orientierte Vergabe knapper Intensivplätze sehr einhellig vorsehen. Hätte das Verfassungsgericht den Gesetzgeber gezwungen, die erfolgsorientierte Priorisierung in Deutschland durch ein neu zu verabschiedendes Gesetz pauschal zu verbieten, so hätte dies den Charakter eines nationalen Alleingangs gehabt.

Zugleich bestätigt das Gericht damit jedoch auch, dass der bisherige regulatorische Umgang mit dem Knappheitsproblem nicht das Potenzial hatte, die berechtigten Sorgen von Menschen mit vorab bestehenden Beeinträchtigungen vollständig zu adressieren. Die häufig geäußerte Feststellung etwa, die einzelnen medizinischen Kriterien würden lediglich darauf abzielen, dass diejenigen behandelt werden, bei denen die größere Aussicht besteht, dass sie durch die Behandlung gerettet werden können^6^, wird für sich genommen sicherlich nicht der Sorge gerecht, die einzelnen medizinischen Kriterien könnten sich dabei dennoch als *indirekt* diskriminierend erweisen. Denn obgleich die empfohlenen Kriterien zur Beurteilung der Erfolgsaussicht nicht *explizit* eine Zurückstellung von Menschen mit konkreten Behinderungen erlauben, steht schließlich zu befürchten, dass die Erfolgsaussichten bei Menschen mit bestimmten Beeinträchtigungen systematisch niedriger sind, so dass sich die empfohlenen Kriterien der Erfolgsbeurteilung als indirekt diskriminierend erweisen würden. Im Grunde schließt dies jedoch auch der neue § 5 des Infektionsschutzgesetzes nicht vollständig aus. Denn Absatz (2) des Paragraphen gestattet eine Berücksichtigung von Komorbiditäten weiterhin, „soweit sie aufgrund ihrer Schwere oder Kombination die auf die aktuelle Krankheit bezogene kurzfristige Überlebensaussicht erheblich verringern“.

Die schwierige Aufgabenstellung des Gerichts, besseren Schutz für Menschen mit Beeinträchtigungen sicherzustellen, muss den Blick auch auf den Umstand lenken, dass in der allgemeinen Bevölkerung sowie auch in der Ethik zum Teil etwas anderes unter einer Diskriminierung verstanden werden könnte als im Recht – ein Umstand, der eine gesamtgesellschaftlich zufriedenstellende Lösung erschweren könnte. Im Folgenden sollen hier einige gängige Positionen aus der philosophischen Ethik in Bezug auf den Diskriminierungsbegriff dargestellt werden. Im Anschluss werden die allgemeinen Zielsetzungen als auch einzelne konkrete Teilkriterien aus der Empfehlung der deutschen medizinischen Fachgesellschaften zum Umgang mit Knappheit auf den Intensivstationen im Lichte dieser ethischen Theoriegruppen auf den Prüfstand gestellt.

Im Ergebnis wird sich dabei zeigen, dass die Frage, ob allgemeine Zielsetzungen oder einzelne Detailkriterien Diskriminierungspotentiale aufweisen, stark davon abhängt, was man unter diesem Begriff zu verstehen hat – eine Frage, auf die mehrere konkurrierende Theoriegruppen unterschiedliche Antworten geben. Des Weiteren wird sich zeigen, dass der mithin engste Ansatz einige Diskriminierungspotentiale nicht zuverlässig aufdecken kann, über die im Grunde Konsens bestehen dürfte. Will man dieses Problem vermeiden, so muss man sich auf breitere Ansätze einlassen, von denen einige wiederum möglicherweise zu viele Handlungen als diskriminierend einstufen. Ein hier genauer untersuchter Ansatz mittlerer Breite lenkt die Aufmerksamkeit auf hilfreiche Weise in Richtung zentraler Anschlussfragen, insbesondere auf das Problem, ob sehr niedrige Wahrscheinlichkeiten eines beträchtlichen Vorteils in Rettungskonflikten als entscheidungsrelevanter Vorteil bewertet werden sollten oder nicht.

Im letzten Teil des Aufsatzes soll das strittige Kriterium der Gebrechlichkeit besprochen werden.^7^ Das Kriterium der Gebrechlichkeit dürfte *bei korrekter Anwendung* im Rahmen aller hier diskutierten Theoriegruppen als unproblematisch eingeschätzt werden – obgleich es in der öffentlichen Debatte mitunter für problematisch gehalten wird und durch die jüngsten Ergänzungen im Infektionsschutzgesetz nun ausgeschlossen wird. Die verschiedenen Theoriegruppen bieten in manchen Fällen also auch überraschend einhelliges Korrekturpotential.

## Theorien der Diskriminierung in der Ethik

Laut einer gängigen Einteilung der verschiedenen ethischen Positionen zu der Frage, wie eigentlich das Übel einer Diskriminierung zu charakterisieren ist, unterscheidet man grob zwischen (i) dem Irrelevanzansatz, (ii) dem schadensbasierten Ansatz und (iii) dem sozialen Bedeutungsansatz (Lippert-Rasmussen 2014; Klonschinski 2020).^8^ In allen drei Theoriegruppen wird davon ausgegangen, dass eine Ungleichbehandlung noch nicht per se eine Diskriminierung darstellt, sondern nur unter bestimmten Bedingungen. Wer zur Einweihungsfeier der neuen Wohnung nur die neue Nachbarschaft, nicht aber das komplette berufliche Umfeld einlädt, behandelt die Personen in den beiden Gruppen zwar ungleich, diskriminiert aber noch nicht per se gegen die Kollegenschaft.^9^

Neben dem Umstand, dass nicht alle Ungleichbehandlungen automatisch Diskriminierungen darstellen, sollte eingangs darauf hingewiesen werden, dass es neben einer Diskriminierung auch noch andere Gründe dafür geben kann, warum eine Ungleichbehandlung problematisch sein kann. Manche Ungleichbehandlungen, die *keine* Diskriminierung darstellen, können demnach in anderer Weise moralisch problematisch sein. (Und natürlich können demnach manche Ungleichbehandlungen auch gleich in mehrfacher Weise moralisch problematisch sein.) Eine Personalchefin beispielsweise, die Bewerberinnen und Bewerber immer nur dann einstellt, wenn deren Unterlagen in einer Vollmondphase bei ihr ankommen, kann vermutlich nicht der Diskriminierung bezichtigt werden, denn ihr Entscheidungskriterium hat nichts mit irgendwelchen Merkmalen der Bewerberinnen und Bewerber zu tun. Aber man kann ihre Entscheidung dennoch kritisieren. So könnte man ihre Ungleichbehandlung der verschiedenen Bewerberinnen nicht nur als irrational und abergläubisch, sondern auch – moralisch wertend – als dreist, achtlos oder faul einschätzen.

In ethischen Besprechungen wird also in der Regel davon ausgegangen, dass nicht alle Ungleichbehandlungen grundsätzlich diskriminierend sind. Manche Ungleichbehandlungen sind überhaupt nicht problematisch und einige andere aus völlig anderen Gründen, die nichts mit einer Diskriminierung zu tun haben. Man will im Rahmen dieser Positionen also darlegen, unter welchen Bedingungen eine Ungleichbehandlung als Diskriminierung gelten sollte.

Im Englischen wird die Möglichkeit moralisch neutraler Ungleichbehandlungen schon auf der Ebene der Wortbedeutung deutlich, denn der Ausdruck „discrimination“ wird häufig auch im Sinne einer moralisch neutralen Unterscheidung oder Differenzierung gebraucht. In englischsprachigen Beiträgen ist daher oft die Rede davon, es müsse geklärt werden, unter welchen Umständen eine Diskriminierung überhaupt moralisch problematisch sei. Im deutschen Sprachgebrauch hingegen wäre dieser moralisch neutrale Gebrauch des Ausdrucks „Diskriminierung“ ungewöhnlich. Bei uns muss die Frage also lauten, unter welchen Umständen eine (möglicherweise moralisch neutrale) Unterscheidung oder Ungleichbehandlung als Diskriminierung gelten sollte.

### Der Irrelevanzansatz

Eine gängige Vorstellung davon, welche Arten von Entscheidungskriterien im Rahmen von Ungleichbehandlungen generell problematisch sind, gibt das deutsche Grundgesetz in Art. 3 Abs. 3:

Niemand darf wegen seines Geschlechtes, seiner Abstammung, seiner Rasse (sic), seiner Sprache, seiner Heimat und Herkunft, seines Glaubens, seiner religiösen oder politischen Anschauungen benachteiligt oder bevorzugt werden. Niemand darf wegen seiner Behinderung benachteiligt werden.

Derartige Listen an Eigenschaften, die als problematische Entscheidungsgrundlagen gelten, kursieren selbstverständlich auch in der Ethik. Doch zugleich gibt es in der ethischen Literatur auch Kritik an der Festlegung auf solche Merkmale. So gibt es Situationen, in denen Merkmale dieser Art entscheidungsrelevant sind und dennoch keine Diskriminierung vorzuliegen scheint: Wenn ein Callcenter beispielsweise nach einer Person sucht, die eine Sprache spricht, in der sie mit der Kundschaft kommunizieren kann, dann stellt dies nach weit verbreiteter Auffassung zunächst einmal keine Diskriminierung dar (trotz Nennung der Sprache in der Liste des Grundgesetzes), sondern den legitimen Wunsch, mit der Kundschaft kommunizieren zu können. Daher ist – auch unter Juristen – häufig zusätzlich von einem sogenannten „entscheidungsrelevanten“ oder „guten Sachgrund“ die Rede, womit gemeint ist, dass eine Andersbehandlung *un*problematisch ist, wenn sie aus guten Gründen erfolgt, zum Beispiel, weil sie für einen an sich legitimen Geschäftserfolg unabdingbar ist. Problematisch werden Entscheidungen aufgrund der genannten Merkmale demnach erst dann, wenn das betreffende Merkmal in einem an sich legitimen Entscheidungskontext nicht zielführend ist und demnach schlicht als irrelevant zu gelten hätte.

Aus ethischer Sicht handelt es sich bei der listenbasierten Konzeption des Grundgesetztes mit der Ausnahmeregelung des relevanten oder guten Sachgrundes am ehesten um den sogenannten Irrelevanzansatz der Diskriminierung (Halldenius 2018). In ethischer Hinsicht ist dieser Ansatz jedoch umstritten (Halldenius 2018; Nickel 1998). Insbesondere besteht der Eindruck, dass das Verhältnis zwischen einer legitimerweise gesuchten Eigenschaft und den dafür verwendeten Indikatoren, bzw. der Frage, ob die verwendeten Indikatoren dafür relevant oder irrelevant sind, nicht zuverlässig anzeigt, ob eine Diskriminierung vorliegt oder nicht. So scheint es zum einen Fälle zu geben, in denen die verwendeten Indikatoren relevant sind, aber dennoch eine Diskriminierung vorliegt. Charakteristisch hierfür sind Szenarien, in denen eine fragwürdige Eigenschaft als „Reaktionsqualifikation“^10^ gesehen werden kann (Wertheimer 1983; Mason 2017). Man denke beispielsweise an einen Kioskbesitzer, der zurecht davon ausgeht, in einer rassistisch gesinnten Gegend tätig zu sein, und nur Personen mit heller Hautfarbe einstellt, weil diese unter seinen Kunden geschäftsdienlichere Reaktionen hervorrufen als Personen mit dunkler Hautfarbe. Unter der Annahme, dass die Menschen in der betreffenden Gegend tatsächlich rassistisch gesinnt sind, ist das Auswahlkriterium des Kioskbesitzers leider relevant, nach gängiger Auffassung liegt aber dennoch eine Diskriminierung vor.^11^

Das noch größere Problem besteht jedoch darin, dass der Irrelevanzansatz keine systematische Aussage darüber trifft, wie eng die Korrelation zwischen einer legitimerweise gesuchten Eigenschaft (z. B. „kann sinnvoll zum Geschäftserfolg des Callcenters beitragen“) und einem dafür verwendeten Indikator (z. B. „spricht Deutsch“, „hat einen Hochschulabschluss“, „verfügt über eine angenehme Stimme“ o. ä.) sein muss, um noch als relevant gelten zu können (Heinrichs 2007). Aus diesem Grund, so könnte man hinzufügen, scheint der Irrelevanzansatz nicht geeignet zu sein, um systematisch Unterscheidungskriterien zu entlarven, die an der Oberfläche zwar unschuldig wirken, aber von vorneherein indirekt diskriminierende Effekte mit sich bringen.

Es ist daher wenig überraschend, dass mitunter argumentiert wird, indirekte Diskriminierungen seien eigentlich gar keine Diskriminierungen.^12^ Um im Rahmen eines Irrelevanz-Ansatzes dennoch Raum für indirekte Diskriminierungen zu finden, kann man die Möglichkeit diskutieren, etwaige disproportionale Effekte zu Ungunsten geschützter Personengruppen zu beachten (Khaitan 2018). Dies erfordert jedoch weitere Äußerungen dazu, wie man disproportionale Nachteile genau bemessen soll (Lippert-Rasmussen 2014, Kap. 2.5) und eine Antwort auf die Frage, unter welchen Umständen es sich bei den disproportionalen Effekten wirklich um eine Kausalfolge des gewählten Proxies handelt und nicht nur um eine zufällige Korrelation.

### Der schadensbasierte Ansatz

Dem schadensbasierten Ansatz zufolge ist eine Ungleichbehandlung dann eine Diskriminierung, wenn sie (a) einer Person aufgrund ihrer Zugehörigkeit zu einer sozial hervortretenden Gruppe^13^ widerfährt und (b) dieser Person dadurch ein Schaden entsteht. Dieser Ansatz wird beispielsweise von Kaspar Lippert-Rasmussen vertreten, der ihn einführend folgendermaßen beschreibt:

It gives special attention to a concept of discrimination that ties discrimination to differential treatment of people on the basis of their membership in socially salient groups. […] it argues that discrimination is wrong first and foremost because of its harmful effects […] (Lippert-Rasmussen 2014, S. 3).

Ähnlich wie der Irrelevanzansatz geht auch diese Position zum einen davon aus, dass Diskriminierungen etwas damit zu tun haben, dass Menschen auf bestimmte Merkmale reduziert werden, die sie als Teil eines ähnlich gearteten Kollektivs erscheinen lassen – obgleich hier keine listenartige Eingrenzung unternommen wird. An die Stelle der Irrelevanzprüfung des vorherigen Ansatzes tritt hier zum anderen jedoch die Prüfung eines mit dieser Eingruppierung verbundenen Schadens.

Von zentraler Bedeutung ist im Rahmen dieses Ansatzes zunächst einmal die Frage, was genau unter einer sozial hervortretenden Gruppe zu verstehen ist. Lippert-Rasmussen charakterisiert eine solche Gruppe folgendermaßen: „Eine Gruppe ist sozial hervortretend, wenn angenommene Mitgliedschaft in dieser Gruppe wichtige Auswirkungen auf die Struktur sozialer Interaktionen in einer Vielzahl sozialer Kontexte mit sich bringt“ (Lippert-Rasmussen 2014, S. 30).^14^ Diese Charakterisierung dürfte die in unserem Grundgesetz genannten Merkmale wie Geschlecht oder Abstammung mit einschließen, ist aber nicht auf diese beschränkt. Ob wichtige Auswirkungen auf das Leben der Betroffenen vorliegen oder nicht, hängt diesem Ansatz zufolge jedoch auch vom soziokulturellen Kontext ab. In einer Gesellschaft wie der unsrigen könnten daher auch Merkmale wie das Rothaarigsein, ärmliche Lebensverhältnisse oder ein ungebildeter Habitus eine sozial hervortretende Gruppe kennzeichnen – das Vorhandensein von grünen Augen hingegen aber eher nicht.

Des Weiteren ist im Rahmen dieses Ansatzes natürlich zu klären, was genau unter einem Schaden zu verstehen ist.^15^ Im Rahmen der Pandemieethik wäre dabei insbesondere die Frage relevant, ob auch die Zumutung eines Risikos oder einer höheren Sterbewahrscheinlichkeit (die dann vielleicht gar nicht zum Tode führt) einen Schaden darstellt. In dieser Hinsicht findet der Ansatz einen gewissen Widerhall in der oben zitierten Grundgesetzpassage, der dem Wortlaut nach natürlich auch noch nicht ohne Weiteres zu entnehmen ist, was genau unter einer „Benachteiligung“ zu verstehen ist.

Zum Umgang mit indirekt diskriminierenden Effekten schlägt Lippert-Rasmussen im Rahmen seines Ansatzes vor, die „Weil-Relation“ oder „Aufgrund-Relation“ so zu gestalten, dass sie auch dann erfüllt ist, wenn es eine überzeugende Kausalerklärung für das Hinnehmen indirekter Effekte ist, dass gegen die betroffene Gruppe bestimmte direkt diskriminierende Gepflogenheiten existieren (Lippert-Rasmussen 2014, S. 38). Ein Unternehmen, in dem die Inanspruchnahme von Elternzeit verpönt ist, könnte demnach zwar argumentieren, dies gälte auch für die männlichen Beschäftigten und nicht nur für die Frauen. Falls dies korrekt wäre, läge tatsächlich keine direkte Diskriminierung vor. Doch die (absichtliche oder auch unabsichtliche) Inkaufnahme des indirekten Effekts, dass dadurch vermutlich sehr viel mehr Frauen benachteiligt werden, dürfte kausal erklärbar sein durch die allgemeine Existenz bestimmter, direkt diskriminierender Haltungen gegen Frauen mit Kindern, die im Berufsleben stehen möchten.

### Der Herabwürdigungsansatz

Dem sozialen Bedeutungsansatz zufolge ist eine Ungleichbehandlung dann problematisch, wenn sie dazu führt, dass eine Gruppe, die sich in einem Machtgefälle zu einer anderen Gruppe befindet, von einem Mitglied der bessergestellten Gruppe auf eine Art und Weise behandelt wird, die das Potenzial einer sozialen Erniedrigung oder Herabwürdigung birgt. Dieser Ansatz wird beispielsweise von Deborah Hellman (2008) vertreten.^16^ Die Frage, ob eine Handlung in diesem Sinne eine herabwürdigende Bedeutung hat, hängt dabei von den historischen und kulturellen Rahmenbedingungen ab und ist insofern objektiv feststellbar. Mit „objektiv“ ist hier gemeint, dass die herabwürdigende Komponente unabhängig davon bestimmt wird, ob die handelnde und die behandelte Person diese Bedeutungskomponente rein subjektiv auch als solche wahrnehmen (Hellman 2008, S. 29, 35). Es handelt sich also – ähnlich wie bei Wortbedeutungen – nicht um eine Einschätzung, bei der irgendwelche involvierten Einzelpersonen die Deutungshoheit haben, sondern vielmehr um eine Bedeutungszuschreibung, die auf breiterer sozialer und historischer Grundlage plausibilisiert sein muss.

Einer allgemeinen Wahrnehmung zufolge ist dieser Ansatz deutlich breiter als andere Positionen. Er kann Ungleichbehandlungen zum Beispiel auch dann noch als diskriminierend einstufen, wenn alle Entscheidungskriterien in Bezug auf das Handlungsziel relevant erscheinen und niemandem unter den betroffenen Personen ein Schaden entsteht. Das heißt: Falls dieser Ansatz korrekt ist, werden deutlich mehr Ungleichbehandlungen als Diskriminierungen zu Buche schlagen als im Rahmen der anderen diskutierten Ansätze.^17^

Ähnlich wie bei der Charakterisierung einer sozial hervortretenden Gruppe im schadensbasierten Ansatz, spielen beim Herabwürdigungsansatz historisch gewachsene soziale Gepflogenheiten bei der Bewertung eine zentrale Rolle. Daher können diese beiden Ansätze das Phänomen der indirekten Diskriminierung recht gut einfangen. Die Einstufung einer Handlung als indirekt diskriminierend hängt schließlich von größeren historischen und sozialen Zusammenhängen ab, nicht von der individuell deklarierten Zielsetzung und Mittelwahl bei einer Handlung. Beim schadensbasierten Ansatz wirken sich soziale Rahmenbedingungen vor allem auf die Einschätzung aus, ob die Betroffenen eine sozial hervortretende Gruppe darstellen oder nicht. Beim Herabwürdigungsansatz hingegen wirken sie sich auf die symbolische Bedeutung der betreffenden Handlung aus, so dass der gesamte Ansatz im Grunde immer auch auf indirekten vermittelten Effekten beruht.

## Formen der Erfolgsorientierung und ihre Diskriminierungspotentiale

Gleich zu Beginn der öffentlichen Debatte um die Empfehlungen der medizinischen Fachgesellschaften für den Umgang mit Knappheitssituationen wurde klar, dass ein effizienzorientiertes Zuteilungssystem verschiedene Formen annehmen kann. Insbesondere lässt sich dabei unterscheiden zwischen (i) der Berücksichtigung des Behandlungserfolgs unter Einbeziehung der längerfristigen Lebenserwartung und (ii) der Berücksichtigung des Behandlungserfolgs ohne Einbeziehung der längerfristigen Lebenserwartung. Im Rahmen der ersten Option zielt man im Grunde darauf ab, mit den begrenzten Ressourcen die größte Anzahl an Lebensmonaten und -jahren zu sichern, während man im zweiten Fall versucht, die größtmögliche Anzahl an Personen zu retten. Im Rahmen der ersten Option – so wurde auch in der öffentlichen Diskussion schnell deutlich – müsste die knappe Ressource im Konfliktfall zwischen Älteren und Jüngeren in der Regel (aber natürlich nicht immer) an die jüngere Person gehen, weil diese im Falle des Behandlungserfolgs dann (zumindest im Allgemeinen) noch eine längere Lebenserwartung hätte. Um diesem Effekt zu entgehen, kann man alternativ auch die kurzzeitige Erfolgsprognose zugrunde legen, also einfach die Wahrscheinlichkeit, mit der eine bedürftige Person durch die Behandlung bis auf Weiteres gerettet werden kann. Auf diese Weise hat man keinen indirekten Verteilungseffekt zu Ungunsten der Älteren und kann zugleich möglichst vielen Menschen erfolgreich helfen.

### Längerfristige Erfolgsaussicht, Alter und Trisomie 21

Obwohl in der Empfehlung der deutschen Fachgesellschaften zunächst sowohl die längerfristige als auch die kurzfristige Erfolgsaussicht eine Rolle spielen sollte, hat man sich ab der zweiten Version des Papiers darauf beschränkt, lediglich die kurzfristige Erfolgsaussicht als Priorisierungsgrundlage zu deklarieren.^18^ Der Grund dafür dürfte insbesondere der geschilderte Effekt der sogenannten Altersdiskriminierung gewesen sein. Auf eine mögliche Priorisierung nach dem Alter soll an dieser Stelle jedoch nicht eingegangen werden, weil sie aus Sicht der Ethik im Detail etwas anders zu beurteilen ist als Priorisierungen aufgrund von in der Regel lebenslang bestehenden Eigenschaften, wie etwa Geschlecht, Herkunft oder Behinderung. Zudem besteht der Auftrag des Bundesverfassungsgerichts nicht darin, Menschen höheren Alters zu schützen, sondern Menschen, die „in der Fähigkeit zur individuellen und selbstständigen Lebensführung längerfristig beeinträchtigt sind“ und deren Beeinträchtigung „von Gewicht“ ist.^19^

Eine etwas weniger augenfällige Auswirkung einer längerfristigen Erfolgsorientierung besteht jedoch darin, dass sie nicht nur ältere Menschen betreffen würde, sondern – beispielsweise – auch Menschen mit Trisomie 21. Denn obgleich die allgemeine Lebenserwartung von Menschen mit Trisomie 21 beträchtlich gestiegen ist, ist sie nach wie vor niedriger als bei Menschen ohne Trisomie (Hithersay et al. 2019; Ng et al. 2017; Presson et al. 2013). Das bedeutet, dass man beim Versuch, mit den begrenzten intensivmedizinischen Ressourcen möglichst viele Lebensmonate und -jahre zu sichern, streng genommen auch Personen mit einer Trisomie 21 hätte zurückstellen müssen, wenn sie mit gleich alten Personen ohne Trisomie (und ansonsten identischen Merkmalen) um einen Platz auf der Intensivstation konkurriert hätten.

Bekannter als im Triage-Kontext ist dieses Problem aus der Debatte um die sogenannten Quality Adjusted Life Years (QALY s) als rechnerische Grundlage für Zuteilungsentscheidungen im Gesundheitswesen. Die Zielsetzung ist dabei, die Effizienz eines Gesundheitswesens zu ermitteln oder sicherzustellen, indem man untersucht, wie sich die investierten Mittel auf die Anzahl an Lebensjahren mit guter Lebensqualität im gesamten System auswirken. Während viele Gesundheitsökonomen mit dieser oder ähnlichen Größen arbeiten und auch einige Krankenversicherungssysteme, wie etwa in Oregon in den USA, sie zeitweilig für Kostenübernahmeentscheidungen angewendet haben, haben Ethiker wie etwa John Harris bereits vor langem darauf hingewiesen, dass es dadurch nicht nur zu Nachteilen für Ältere, sondern auch zu Benachteiligungen aufgrund von Behinderungen kommen kann (Harris 1987).

### Diskriminierungspotentiale der längerfristigen Erfolgsaussicht bei Trisomie 21

An dieser Stelle soll nun der Bogen geschlagen werden zu den verschiedenen Theorien der Diskriminierung, wie sie in der Ethik diskutiert werden. Beginnen wir dabei wieder mit dem Irrelevanzansatz, demzufolge eine Ungleichbehandlung immer dann eine Diskriminierung darstellt, wenn bei Entscheidungen ein für die eigentliche Zielsetzung irrelevantes Merkmal von Personengruppen zugrunde gelegt wird. Die Frage ist nun die folgende: Würde der Irrelevanzansatz die Zielsetzung, längerfristige Behandlungserfolge zu gewährleisten, als problematisch einstufen? Der Gedanke, Menschen mit Trisomie 21 aufgrund geringerer Lebenserwartung, die noch dazu mit ihrer Beeinträchtigung zusammenhängen dürfte, zurückzustellen, erscheint besorgniserregend. Doch lässt sich dieser Eindruck im Rahmen des Irrelevanzansatzes begründen?

Tatsächlich ist es fraglich, ob der übliche Irrelevanzansatz hier ein Problem erkennen kann. Erstens kann ein Rekurs auf die gängigen Listen an Merkmalen, die dem Irrelevanzansatz zufolge regelhaft als problematisch zu gelten haben (Geschlecht, Herkunft, Behinderung, Religion ect.), das Problem nicht ohne Weiteres erfassen. Denn in medizinischen Handlungsempfehlungen wird ja grundsätzlich nicht stehen, dass Menschen mit Behinderungen oder speziell Menschen mit einer Trisomie 21 im Knappheitsfall zurückzustellen seien. Zu finden sein dürfte in derartigen Quellen höchstens der Hinweis, die längerfristige Erfolgsaussicht sei zu beachten. Diese Forderung ist aber nicht deckungsgleich mit der Forderung, Menschen mit Trisomie 21 seien zurückzustellen. Denn offenkundig gibt es viele Menschen mit Trisomie 21, die noch eine sehr lange Lebenserwartung haben. Und offenkundig gibt es auch viele Menschen mit Trisomie 21, die noch eine längere Lebenserwartung haben als andere Menschen, die ebenfalls eine Intensivbehandlung benötigen könnten. Unter diesen anderen Bedürftigen würden sich realistisch betrachtet auch viele Ältere mit weit fortgeschrittenen, schwerwiegenden Vorerkrankungen befinden. Und in solchen Fällen würde eine Zurückstellung von Menschen mit Trisomie 21 aufgrund der längerfristigen Lebenserwartung gerade *nicht* erfolgen. Eine Zurückstellung von Menschen mit Trisomie 21 wäre somit *keine notwendige Folge* der Beachtung längerfristiger Lebenserwartung, sondern lediglich eine Art von *potenzieller, aber strukturell erwartbarer Folge *der Forderung, die längerfristige Erfolgsaussicht zu beachten. Wir hätten es also wohl mit dem bereits oben geschilderten Problem zu tun, dass der Irrelevanzansatz das Phänomen der indirekten Diskriminierung nicht optimal erfassen kann.

Zweitens besteht im Rahmen dieses Ansatzes das notorische Problem, dass behauptet werden kann, die Trisomie sei eben ein relevanter Indikator für die längerfristige Lebenserwartung und damit im Allgemeinen ein guter Sachgrund für eine Zurückstellung im Rahmen eines Systems, das auf längerfristige Erfolgsaussichten abstellt. Der Kern des Ansatzes ist die von vorneherein zielbezogene Relevanzprüfung des gewählten Entscheidungsmerkmals. In anderen Worten: Es geht um die rein instrumentelle Frage, ob das ausgesuchte Proxy (hier: „hat Trisomie 21“) im Hinblick auf ein an sich legitim wirkendes Handlungsziel (hier: „längerfristige Behandlungserfolge erzielen“) einen sachlich guten Entscheidungsgrund darstellt. Das Ziel, längerfristige Behandlungserfolge zu erzielen, dürfte an der Oberfläche betrachtet vielen Menschen legitim erscheinen. Durch die rein instrumentelle Zweckmäßigkeitsprüfung, die der Irrelevanzansatz im Hinblick auf dieses scheinbar legitime Ziel vornimmt, ist das Problem an sich daher nicht zuverlässig zu erkennen.

Möglicherweise könnte an dieser Stelle eingewandt werden, der Irrelevanzansatz könne durchaus dazu herangezogen werden, zu argumentieren, dass eine Trisomie 21 und die etwas kürzere Lebenserwartung der Betroffenen in derartig engem Zusammenhang miteinander stünden, dass eine Zurückstellung aufgrund der niedrigeren Lebenserwartung einer Zurückstellung aufgrund der Trisomie gleichkomme. Doch ist das wirklich der Fall? Studien belegen einen starken Anstieg der Lebenserwartung von Menschen mit Trisomie 21 in bestimmten Ländern in den vergangenen Jahrzehnten (Hithersay et al. 2019; Ng et al. 2017; Presson et al. 2013). Dieser Umstand scheint nahezulegen, dass die Lebenserwartung der Betroffenen eher von der Qualität der medizinischen Versorgung abhängt als von ihrer genetischen Ausstattung. Der Zusammenhang zwischen Lebenserwartung und Genetik ist also möglicherweise weniger zwingend als mitunter vielleicht angenommen wird. Und – wie oben ebenfalls bereits erläutert – macht der Irrelevanzansatz leider keinerlei Aussage darüber, wie eng die Korrelation zwischen einer Zielgröße und einem als Entscheidungsgrundlage verbotenen Merkmal sein muss, um auch die Zielgröße als problematisch erscheinen zu lassen. Man scheint also auf bloße Intuitionen zurückgeworfen zu sein. Man könnte das Problem auch folgendermaßen ausdrücken: Ohne eine Ergänzung über die noch erlaubte Korrelationsdichte zwischen angestrebter Zielsetzung und dafür verwendetem Proxy bietet der Ansatz vermutlich keine gute Grundlage für eine Beurteilung mutmaßlicher indirekter Diskriminierungen.

Auf alleiniger Grundlage des Irrelevanzansatzes der Diskriminierung lässt sich ein etwaiges Problem hier also gar nicht sicher erfassen. Besser ist es daher vielleicht, auf die zusätzlichen und breiteren Ansätze der Diskriminierung, wie etwa den schadensbasierten Ansatz und den sozialen Bedeutungsansatz zurückzugreifen. Im Rahmen des schadensbasierten Ansatzes könnte die Analyse in knapper Form (mehr dazu später) ungefähr folgendermaßen aussehen: Es ist stark davon auszugehen, dass Menschen mit Trisomie 21 eine sozial hervortretende Gruppe bilden. Es dürfte unstrittig sein, dass eine solche unfreiwillige Zurückstellung im Knappheitsfall ein Schaden oder ein Nachteil wäre. Und im Rahmen des sozialen Bedeutungsansatzes könnte darüber hinaus argumentiert werden, dass es eine entsprechend problematische Bedeutungskomponente hätte, eine Person aus der historisch benachteiligten Gruppe der Menschen mit Trisomie 21 auf die Palliativstation zu bringen, während eine andere Person mit ansonsten identischen Eigenschaften auf die Intensivstation gebracht wird und die notwendige Hilfe erhält. Diese etwas breiteren Ansätze diagnostizieren das Problem hier also zuverlässiger.

## Konkrete medizinische Kriterien und ihre Diskriminierungspotentiale

Das Abzielen auf konkrete Formen des Behandlungserfolgs – kurzfristiger oder längerfristiger Art – kann nur auf Grundlage von Erfahrungswerten erfolgen. Zu Beginn einer Pandemie werden solche Erfahrungswerte auf recht allgemeinen Kriterien beruhen und daher noch etwas spekulativer sein. Sobald bessere Daten vorhanden sind, werden Prognosen verlässlicher. Die deutsche Empfehlung für den Umgang mit Knappheit auf den Intensivstationen entstand in einem relativ frühen Stadium der Pandemie und deutet in den ersten beiden Version recht unumwunden an, dass viele der medizinischen Kriterien, die dort als aussagekräftig für die Erfolgsaussicht dargestellt werden, keine COVID-spezifischen prognostischen Marker darstellen (DIVI et al. 2020a, b, 2021, S. 8). Dennoch – soviel darf man den Medizinerinnen und Medizinern vermutlich zugestehen – geben auch diese medizinischen Kriterien in sehr vielen anderen Fällen gut begründeten Anlass zu der Annahme, dass eine Erfolgsaussicht schlecht oder gar nicht mehr vorhanden ist.

### Die Relevanz der „fortgeschrittenen Lungenerkrankung“ und die Mukoviszidose

Die Empfehlung der Fachgesellschaften gibt eine Reihe von Kriterien für eine schlechte Erfolgsaussichten an, die im Knappheitsfall zu einer nachrangigeren Behandlungspriorität führen sollen. Dazu zählen nicht nur der Schweregrad der „führenden Erkrankung“, sondern auch etwaige Komorbiditäten sowie der später noch zu besprechende Gebrechlichkeitsgrad der betroffenen Person. Zu den relevanten Komorbiditäten gehören insbesondere schwere „Organ-Dysfunktionen mit prognostisch eingeschränkter Lebenserwartung“, wie beispielsweise eine „fortgeschrittene Lungenerkrankung“ (DIVI et al. 2020a, b, 2021, S. 8).

Untersuchen wir nun die Diskriminierungspotentiale eines Kriteriums wie der „fortgeschrittenen Lungenerkrankung“. Behindertenvertreterinnen und -vertreter könnten hier die zunächst einmal verständliche Sorge äußern, dass Menschen mit bestimmten Beeinträchtigungen häufiger an einer fortgeschrittenen Lungenerkrankung leiden als andere. Zu denken wäre hier beispielsweise an Personen mit Mukoviszidose, einer angeborenen, genetisch bedingten Erkrankung oder Behinderung, die sich aufgrund einer Verdickung des Sekrets in den Bronchien negativ auf die Lungenfunktion auswirkt und in der Regel frühzeitig zum Tode führt.

Nehmen wir nun eine Person mit *fortgeschrittener* Mukoviszidose. Die Empfehlung der deutschen Fachgesellschaften besagt, dass diese Person aufgrund ihrer fortgeschrittenen Lungenprobleme geringere Erfolgsaussichten aufweist und daher im Knappheitsfalle zurückgestellt werden sollte, um die knappen Ressourcen für Personen einzusetzen, bei denen die Rettung aussichtsreich erscheint. Gehen wir des Weiteren davon aus, dass fortgeschrittene Lungenprobleme das Überstehen einer zusätzlichen COVID-19-Erkrankung höchstwahrscheinlich tatsächlich ernsthaft gefährden.

Unter der kausalen Vorbedingung, dass die fortgeschrittene Lungenerkrankung für das Ziel einer erfolgreichen Rettung relevant ist, würde der Irrelevanzansatz der Diskriminierung hier kein Problem erkennen. Ein Problem bestünde laut Irrelevanzansatz dann, wenn in der Empfehlung der Fachgesellschaften stünde, dass alle Personen *mit Mukoviszidose* zurückgestellt werden sollen. Das steht dort aber nicht. Es ist dort lediglich von fortgeschrittenen Lungenerkrankungen die Rede. Manchen Mukoviszidose-Patienten geht es ja recht gut, so dass ihre Überlebensaussichten dann vermutlich gerade nicht besonders eingeschränkt wären. Die in der Empfehlung getroffene Aussage ist spezifischer und impliziert, dass nur diejenigen Mukoviszidoseerkrankungen, die sich in fortgeschrittenem Stadium befinden, zu einer Zurückstellung führen sollen.

### Schadensbasierter Ansatz und Herabwürdigungsansatz zur Mukoviszidose

Wie sähe diese Situation aus der Perspektive des Schädigungsansatzes der Diskriminierung aus? Auch der Schadensansatz hat zwei Teile: Problematisch sind demnach Ungleichbehandlungen aufgrund der (i) Zugehörigkeit zu einer sozial hervortretenden Gruppe, aufgrund derer (ii) den Betroffenen ein Schaden oder Nachteil entsteht. Wird durch das Kriterium der fortgeschrittenen Lungenerkrankung Personen mit Mukoviszidose aufgrund ihrer Zugehörigkeit zu einer sozial hervortretenden Gruppe ein Schaden oder Nachteil zugemutet?

In Bezug auf die erste Hälfte des Ansatzes kann man sich vermutlich streiten. Sozial hervortretend im Sinne von Lippert-Rasmussen wäre die Mukoviszidose dann, wenn die angenommene Mitgliedschaft in dieser Gruppe für die Betroffenen in einer Vielzahl von Lebensbereichen die Struktur ihrer sozialen Interaktionen maßgeblich beeinflusst. Vermutlich hinge die Antwort davon ab, in wie vielen Lebensbereichen sich Kernelemente der sozialen Interaktionen auf die Mukoviszidose zurückführen lassen.

Der zweite Teil des schadensbasierten Ansatzes besagt, dass aufgrund dieser Gruppenzugehörigkeit ein Schaden oder ein Nachteil entstanden sein muss. Hier wäre man zunächst geneigt, das Definitionsmerkmal erfüllt zu sehen, weil es auf den ersten Blick ein beträchtlicher Nachteil sein dürfte, im Falle höchster Not nicht mehr in den Genuss eines Rettungsversuchs zu gelangen. Wie sieht es aber aus, wenn man die Wahrscheinlichkeiten in die Nutzenbetrachtung mit einbezieht? Falls fortgeschrittene Lungenerkrankungen tatsächlich schlechtere Erfolgsaussichten bedeuten, müsste dies auch für eine fortgeschrittene Mukoviszidose gelten, und die Wahrscheinlichkeit, die Erkrankung mittels Behandlung zu überstehen, müsste für Personen mit einem solchen Leiden wesentlich geringer sein. Das bedeutet, betroffene Personen würden die Erkrankung auch mit einer Behandlung mit recht hoher Wahrscheinlichkeit nicht überstehen. Das bedeutet auch: Die Wahrscheinlichkeit, dass diese Personen hier einen Nachteil haben, wenn sie nicht behandelt werden, ist für diese Personen geringer als für andere Personen mit besseren Aussichten. Technisch gesprochen stellt sich hier die Frage, ob man den möglichen ex post-Nutzen (Lebensrettung) für entscheidend halten soll oder vielmehr den ex ante-Nutzen, also das Nutzenpotential im Zusammenspiel mit dessen Eintrittswahrscheinlichkeit (Wahrscheinlichkeit der Lebensrettung). Unbenommen von der Möglichkeit jedoch, dass man behaupten kann, der ex ante-Schaden durch Nicht-Behandlung wäre für Personen mit fortgeschrittener Mukoviszidose geringer als für Menschen mit besseren Erfolgsaussichten, lässt sich nicht leugnen, dass es sich auch bei einem geringeren derartigen Schadenspotential noch immer um ein Schadenspotential handelt.

Die Frage, ob der schadensbasierte Ansatz hier eine Diskriminierung feststellt, hängt also von der Einschätzung ab, ob Menschen mit Mukoviszidose eine sozial hervortretende Gruppe bilden oder nicht. Denn die Schadenskomponente des Ansatzes wäre für sich genommen erfüllt. Die Frage ist im Rahmen dieses Ansatzes also, ob für Betroffene die sozialen Interaktionen aufgrund angenommener Zugehörigkeit zur Gruppe der Mukoviszidosepatienten generell maßgeblich beeinträchtigt werden.

Den weitreichendsten Spielraum könnte eventuell wieder der Herabwürdigungsansatz bieten, dessen Auswirkungen hier nur kurz umrissen werden sollen. Er würde die zusätzliche Frage aufwerfen, ob es objektiv eine erniedrigende Bedeutungskomponente hat, einer Person mit fortgeschrittener Mukoviszidose eine Behandlung vorzuenthalten. Es ist nicht ganz einfach, diese Frage zu beantworten. Möglicherweise muss man sie in Abhängigkeit von der historischen und aktuellen Situation der Mukoviszidose-Patientinnen und -Patienten beantworten. Wenn wir uns als Sozialgesellschaft nicht genügend um die Belange dieser Personengruppe kümmern, und beispielsweise schon ihre Regelversorgung notorisch schlecht ist, könnte eine Zurückstellung im Knappheitsfalle möglicherweise als symbolhafter Hinweis auf eben diesen generell illegitimen Umstand interpretiert werden. Falls die Frage der Herabwürdigung also auf diese oder auf eine andere Weise mit Ja beantwortet werden muss, so müsste man auf Grundlage dieses Ansatzes davon ausgehen, dass das Kriterium der fortgeschrittenen Lungenerkrankung eine Diskriminierung von Menschen mit Beeinträchtigungen wie einer genetisch bedingten Lungenerkrankung darstellt.

Es kann durchaus argumentiert werden, dass Menschen mit Mukoviszidose *keine* sozial hervortretende Gruppe bilden. In breiten Teilen der Gesellschaft dürfte beispielsweise unbekannt sein, was die Mukoviszidose ist – so dass auch keine speziellen Vorannahmen über Menschen mit Mukoviszidose vorliegen dürften. Zudem sind diese Patientinnen und Patienten in den meisten Kontexten nicht ohne weiteres von Menschen ohne Mukoviszidose zu unterscheiden, was es unwahrscheinlich macht, dass sie häufig anders behandelt werden. Diese Umstände sprechen gegen eine Einstufung als sozial hervortretende Gruppe. Aus ähnlichen Gründen kann im Rahmen des Herabwürdigungsansatzes bestritten werden, dass das Kriterium der fortgeschrittenen Lungenerkrankung eine sozialhistorisch objektivierbare Bedeutungskomponente aufweist, die Menschen mit Mukoviszidose herabwürdigt. Diese Überlegungen lassen den Schluss zu, dass hier auch aus Sicht der beiden zuletzt diskutieren Theorien keine direkte Diskriminierung vorliegt. Bei anderen Behinderungen können derartige Einschätzungen natürlich anders ausfallen.

### Indirekte Diskriminierung und die Mukoviszidose

Wie sieht das Analyseergebnis nun aber aus, wenn nicht gefragt wird, ob eine direkte Diskriminierung vorliegt, sondern eine *indirekte* Diskriminierung?

(i) Beim Irrelevanzansatz muss man sich hierzu im Grunde genommen erst einmal auf eine konkrete Zusatztheorie zur indirekten Diskriminierung festlegen. Wie oben angedeutet, ist dieser Ansatz im Prinzip kompatibel mit der Auffassung, dass es indirekte Diskriminierungen gar nicht gibt. Will man ihn um eine entsprechende Theoriekomponente ergänzen, so kommt die Konstruktion der disproportionalen Benachteiligungen in Betracht. Die Frage wäre demnach, ob das Priorisierungskriterium der fortgeschrittenen Lungenerkrankung für Personen mit Mukoviszidose in disproportionaler Weise zu Nachteilen führt. Dazu muss zunächst einmal der Anteil der Personen mit Mukoviszidose unter denjenigen ermittelt werden, die von der Regelung benachteiligt werden. Anschließend muss dieser Anteil verglichen werden mit dem Anteil der Mukoviszidosepatienten unter denjenigen, die von der Regelung profitieren. Da viele – und vermutlich die meisten – Mukoviszidosepatienten gerade nicht in fortgeschrittenem Stadium erkrankt sind, führt diese Analyse womöglich zu dem Ergebnis, dass Mukoviszidosepatienten disproportional von der Regelung *profitieren*. Von einer indirekten Diskriminierung könnte in diesem Fall dann keine Rede sein.

(ii) Im Rahmen des schadensbasierten Ansatzes wird – wie oben erläutert – vorgeschlagen, immer dann von einer indirekten Diskriminierung auszugehen, wenn die indirekten Effekte einer Handlung oder Regelung kausal darauf zurückgeführt werden können, dass gegen die betroffene Gruppe auch direkt diskriminierende Gepflogenheiten existieren. Auf diese Weise sollen indirekt diskriminierende Effekte von bloßen Korrelationen unterschieden werden. Das Priorisierungskriterium der fortgeschrittenen Lungenerkrankung würde demnach also nur dann als indirekte Diskriminierung gegen Menschen mit Mukoviszidose gelten, wenn gezeigt werden könnte, dass es direkte Diskriminierungen gegen diese Menschen gibt und die Wahl des Kriteriums der fortgeschrittenen Lungenerkrankung durch diese direkt diskriminierenden Haltungen verursacht wurde.

(iii) Im Rahmen des Herabwürdigungsansatzes kann man davon ausgehen, dass gewissermaßen alle diskriminierenden Handlungen einen indirekten (Teil‑)Charakter haben, weil die herabwürdigende Bedeutung von Handlungen erst durch den unabhängig existierenden gesellschaftlichen Rahmen entsteht, in dem sie stattfinden. Die Analysen, die anzustellen sind, um herauszufinden, ob eine indirekte Diskriminierung vorliegt, werden sich bei diesem Ansatz von den Untersuchungen zur Ermittlung direkter Diskriminierungen daher kaum unterscheiden.

Es kann also nicht ohne Weiteres davon ausgegangen werden, dass das Priorisierungskriterium der fortgeschrittenen Lungenerkrankung als *indirekte* Diskriminierung von Menschen mit Mukoviszidose zu werten ist. Dieser Skizzierung zufolge scheint keine indirekte Diskriminierung vorzuliegen. Es sei jedoch darauf hingewiesen, dass diese Schematisierung nicht den Anspruch einer vollständigen und endgültigen Analyse erhebt und es sei noch einmal daran erinnert, dass derartige Einschätzungen bei anderen Behinderungen völlig anders ausfallen können.

## Schluss

Insgesamt, so kann man sagen, hängt die Beurteilung der Frage, ob in konkreten Fällen moralisch problematische Diskriminierungen aufgrund von Behinderungen vorliegen, häufig davon ab, welche genaue Theorie der Diskriminierung man vertritt. Dadurch kann natürlich die Situation entstehen, dass einige Kommentatoren eine Diskriminierung sehen, während andere das abstreiten. Die öffentliche Debatte über solche Sachverhalte könnte an Differenzierung gewinnen, wenn man sich genauer über die verschiedenen Grundlagen einer Diskriminierungsbehauptung unterhalten würde. Während der vergleichsweise enge Irrelevanzansatz für sich genommen nicht alle Phänomene erfassen kann, haben die breiteren Ansätze den Nachteil, komplexere soziokulturelle Erwägungen mit einbeziehen zu müssen.

Ein weiteres wichtiges Analyseergebnis dieser Arbeit dürfte darin bestehen, dass der Irrelevanzansatz für sich genommen das Phänomen indirekter Diskriminierungen nicht klar genug zu fassen bekommt, um die Sorgen der Menschen mit Behinderungen in Bezug auf die Empfehlung der deutschen Fachgesellschaften angemessen zu adressieren. Um diese Aufgabe zu erfüllen, muss er um weitere Theoriekomponenten ergänzt werden. Der Herabwürdigungsansatz hingegen erscheint vergleichsweise breit zu sein und dürfte Ungleichbehandlungen daher im größten Umfang als Diskriminierungen einstufen. Am differenziertesten erscheint vor diesem Hintergrund das Analysepotential des schadensbasierten Ansatzes, der den Blick auf die Frage lenkt, in welchem Umfang soziale Gepflogenheiten gegenüber betroffenen Personen die Struktur ihrer sozialen Interaktionen beeinträchtigen.

## Footnotes

1 BVerfG, 1 BvR 1541/20, 16.12.2021, Abs. 87.

2 BVerfG, 1 BvR 1541/20, 16.12.2021, Abs. 86, 87, 88, 96 u. 97.

3 BVerfG, 1 BvR 1541/20, 16.12.2021, Abs. 116. Ausführliche ethische Besprechungen der Legitimität einer erfolgsorientierten Allokation, die jedoch nicht genauer auf die verschiedenen Theorien der Diskriminierung eingehen, liefern u. a. die Beiträge von Dieter Birnbacher und Weyma Lübbe in Hörnle et al. (2021).

4 U. a. BVerfG, 1 BvR 1541/20, 16.12.2021, Abs. 117.

5 Einige Beispiele: Australian and New Zealand Intensive Care Society (ANZICS 2021); Belgian Society of Intensive Care Medicine (SIZ 2020); British Medical Association (BMA 2020); Canadian Medical Association (CMA 2020); German Interdisciplinary Association of Intensive Care and Emergency Medicine (DIVI et al. 2020c); Italian Society for Anesthesia, Analgesia, Emergency and Intensive Care Medicine (SIAARTI 2020); für die Niederlande: Verweij M et al. (2020); Swiss Academy of Medical Sciences (SAMW 2020); Critical Care Society of Southern Africa (CCSSA 2020); in den Vereinigten Staaten: Maves et al. (2020).

6 Immer wieder betont beispielsweise in Pressemitteilungen der Deutsche Interdisziplinäre Gesellschaft für Intensiv- und Notfallmedizin (DIVI) und Stellungnahmen der Autoren der Empfehlung. Siehe auch BVerfG, 1 BvR 1541/20, 16.12.2021, Abs. 118.

7 Die ebenfalls strittige Frage der sogenannten Ex-Post-Triage bedarf einer Besprechung in anderem Theorierahmen als dem Diskriminierungskontext.

8 Lippert-Rasmussen charakterisiert den Irrelevanz-Ansatz als eine Variante der Mental-State-Based Accounts.

9 Diejenigen, die dieses Beispiel diskutabel finden, stellen sich bitte zusätzlich vor, dass die neue Wohnung in einem hinreichend heterogenen Stadtviertel liegt.

10 Hierbei handelt es sich um eine Übersetzung des englischen Ausdrucks „reaction qualification“.

11 Auch der umgekehrte Fall scheint zu existieren. Es scheint Fälle zu geben, in denen das gewählte Entscheidungskriterium tatsächlich irrelevant ist, aber zugleich dennoch keine Diskriminierung vorliegt. Man denke hier an das Beispiel der Personalchefin, die lediglich in Vollmondphasen einstellt.

12 So zum Beispiel Cavanagh (2002), Eidelson (2015), Young (1990) oder überblicksartig Altmann (2020, Abs. 3.1), die zugunsten dieser Schlussfolgerung natürlich auch noch weitere Argumente anführen.

13 Im englischen Original verwendet Lippert-Rasmussen den Ausdruck „socially salient group“, der manchmal auch als „sozial auffällige Gruppe“ übersetzt wird. Der von vorneherein stark negative Charakter dieser alternativen Übersetzung soll hier jedoch vermieden werden.

14 In dieser Hinsicht ähnelt der Ansatz einer vorherigen Ausarbeitung von Radcliffe Richards (1985, S. 66–75).

15 Abweichend vom Grundgesetz sieht der schadensbasierte Ansatz in der Ethik offenkundig nicht vor, dass auch Bevorzugungen eine Diskriminierung darstellen können. Wenn eine Bevorzugung der einen Person oder Gruppe mit keinerlei Nachteil für eine andere Person oder Gruppe einhergeht, so würde dem schadensbasierten Ansatz zufolge auch nirgends eine Diskriminierung stattfinden. Laut Grundgesetz wäre das bei Vorliegen der dort genannten Entscheidungsmerkmale jedoch der Fall. Diese Asymmetrie zwischen dem schadensbasierten Ansatz und dem Grundgesetzparagraphen soll an dieser Stelle aber nicht weiterverfolgt werden, weil in Knappheitssituationen auf Intensivstationen mit einem Behandlungsvorteil für die eine Person notwendigerweise ein Nachteil für eine andere Person einhergeht.

16 Ein verwandter Ansatz besagt zudem, das Problem der Diskriminierung sei die Respektlosigkeit (Eidelson 2015).

17 Die hier diskutierten Theorien geben zwar vor, lediglich die Frage zu beantworten, worin das Übel der Diskriminierung genau besteht. Doch interessanterweise wirken sie sich auch auf die hierbei zugrunde liegende Primäreinschätzung darüber aus, welche Handlungen überhaupt als Diskriminierungen zu Buche schlagen sollen. Diese Überschneidung zweier an sich separater Fragestellungen macht die Debatte häufig nicht gerade einfacher.

18 Während in Version 1 der deutschen Empfehlung noch vorgesehen war, diejenigen Personen vorrangig zu behandeln, bei denen „eine bessere Gesamtprognose (auch im weiteren Verlauf)“ vorliegt, ist ab Version 2 nur noch „eine höhere Überlebenswahrscheinlichkeit“ ausschlaggebend.

19 BVerfG, 1 BvR 1541/20, 16.12.2021, Abs. 90.

## References

[CR1] Altmann A, Zalta E (2020). Discrimination. Stanford encyclopaedia of philosophy.

[CR2] Australian and New Zealand Intensive Care Society (ANZICS) (2021) Guiding principles for complex decision making during Pandemic COVID-19. https://www.anzics.com.au/wp-content/uploads/2021/09/ANZICS-COVID-19-Guidelines-Version-4.pdf. Zugegriffen: 10. Sept. 2022

[CR3] Belgian Society of Intensive Care Medicine (2020) Ethical principles concerning proportionality of critical care during the 2020 COVID-19 pandemic in Belgium: Advice by the Belgian Society of Intensive Care Medicine. https://www.siz.be/wp-content/uploads/COVID_19_ethical_E_rev3.pdf. Zugegriffen: 10. Sept. 2022

[CR4] British Medical Association (BMA) (2020) COVID-19—Ethical issues. A guidance note. https://www.bma.org.uk/media/2360/bma-covid-19-ethics-guidance-april-2020.pdf. Zugegriffen: 10. Sept. 2022

[CR5] Canadian Medical Association (CMA) (2020) Framework for ethical decision making during the coronavirus pandemic. https://policybase.cma.ca/en/permalink/policy14133. Zugegriffen: 10. Sept. 2022

[CR6] Cavanagh M (2002). Against equality of opportunity.

[CR7] Critical Care Society of Southern Africa (CCSSA) (2020) Allocation of scarce critical care resources during the COVID-19 public health emergency in South Africa. https://criticalcare.org.za/wp-content/uploads/2020/05/V2-2020-May-05-Allocation-of-Scarce-Critical-Care-Resources-During-the-COVID-19-Public-Health-Emergency-in-South-Africa-FINAL-.pdf. Zugegriffen: 10. Sept. 2022 32880283

[CR8] Deutsche Interdisziplinäre Vereinigung für Intensiv- und Notfallmedizin (DIVI) et al (2020a) Entscheidungen über die Zuteilung intensivmedizinischer Ressourcen im Kontext der COVID-19-Pandemie, Version 1. https://www.divi.de/joomlatools-files/docman-files/publikationen/covid-19-dokumente/200325-covid-19-ethik-empfehlung-v1.pdf. Zugegriffen: 10. Sept. 2022

[CR9] Deutsche Interdisziplinäre Vereinigung für Intensiv- und Notfallmedizin (DIVI) et al (2020b) Entscheidungen über die Zuteilung intensivmedizinischer Ressourcen im Kontext der COVID-19-Pandemie, Version 2. https://www.divi.de/empfehlungen/publikationen/viewdocument/3436/covid-19-ethik-empfehlung-v2. Zugegriffen: 10. Sept. 2022

[CR10] Deutsche Interdisziplinäre Vereinigung für Intensiv- und Notfallmedizin (DIVI) et al (2020c) Decisions on the allocation of intensive care resources in the context of the COVID-19 pandemic. https://pubmed.ncbi.nlm.nih.gov/32728769/. Zugegriffen: 10. Sept. 2022

[CR11] Deutsche Interdisziplinäre Vereinigung für Intensiv- und Notfallmedizin (DIVI) et al (2021) Entscheidungen über die Zuteilung intensivmedizinischer Ressourcen im Kontext der COVID-19-Pandemie, Version 3. https://register.awmf.org/assets/guidelines/040-013l_S1_Zuteilung-intensivmedizinscher-Ressourcen-im-Kontext-der-COVID-19-Pandemie-Klinisch-ethische_Empfehlungen_2021-12_1.pdf. Zugegriffen: 10. Sept. 2022

[CR12] Eidelson B (2015). Discrimination and disrespect.

[CR13] German Interdisciplinary Association of Intensive Care and Emergency Medicine (2021). Decisions on the allocation of intensive care resources in the context of the COVID-19 pandemic. Med Klin Intensivmed Notfmed.

[CR15] Halldenius L, Lippert-Rasmussen K (2018). Discrimination and irrelevance. Routledge handbook of the ethics of discrimination.

[CR14] Harris J (1987). QALYfying the value of life. J Med Ethics.

[CR16] Heinrichs B (2007). What is discrimination and when is it morally wrong?. JWE.

[CR17] Hellman D (2008). When is discrimination wrong?.

[CR18] Hithersay R, Startin CM, Hamburg S, Mok KY, Hardy J, Fisher EMC, Tybulewicz VLJ, Nizetic D, Strydom A (2019). Association of dementia with mortality among adults with down syndrome older than 35 years. JAMA Neurol.

[CR19] Hörnle T, Huster S, Poscher R (2021). Triage in der Pandemie.

[CR20] Italian Society for Anesthesia, Analgesia, Emergency and Intensive Care Medicine (SIAARTI) (2020) Clinical ethics for the allocation of intensive care treatments. https://ccforum.biomedcentral.com/articles/10.1186/s13054-020-02891-w. Zugegriffen: 10. Sept. 2022

[CR21] Khaitan T, Lippert-Rasmussen K (2018). Indirect discrimination. Routledge handbook of the ethics of discrimination.

[CR22] Klonschinski A (2020). Was ist Diskriminierung und was genau ist daran moralisch falsch? Einleitung zum Themenschwerpunkt Diskriminierung. ZfpP.

[CR23] Lippert-Rasmussen K (2014). Born free and equal? An inquiry into the nature of discrimination.

[CR24] Mason A (2017). Appearance, discrimination, and reaction qualification. J Polit Philos.

[CR25] Maves RC, Downar J, Dichter JR, Hick JL, Devereaux A, Geiling JA, Kissoon N, Hupert N, Niven AS, King MA, Rubinson LL, Hanfling D, Hodge JG Jr., Marshall MF, Fischkoff K, Evans LE, Tonelli MR, Wax RS, Seda G, Parrish JS, Truog RD, Sprung CL, Christian MD (2020) Triage of scarce critical care resources in COVID-19. An implementation guide for regional allocation: An Expert Panel Report of the Task Force for Mass Critical Care and the American College of Chest Physicians. https://pubmed.ncbi.nlm.nih.gov/32289312/. Zugegriffen: 10. Sept. 2022 10.1016/j.chest.2020.03.063 PMC7151463 32289312

[CR26] Ng N, Flygare Wallén E, Ahlström G (2017). Mortality patterns and risk among older men and women with intellectual disability: a Swedish national retrospective cohort study. BMC Geriatr.

[CR27] Nickel JW, Craig E (1998). Discrimination. Routledge encyclopedia of philosophy.

[CR28] Presson AP, Partyka G, Jensen KM, Devine OJ, Rasmussen SA, McCabe LL, McCabe ER (2013). Current estimate of down syndrome population prevalence in the United States. J Pediatr.

[CR29] Richards R (1985). Discrimination. Proc Aristot Soc.

[CR31] Swiss Academy of Medical Sciences (SAMW) (2020) COVID-19 pandemic: Triage for intensive-care treatment under resource scarcity, Version 3. https://smw.ch/article/doi/smw.2020.20229. Zugegriffen: 10. Sept. 2022 10.4414/smw.2020.20229 32208495

[CR32] Verweij M, van de Vathorst S, Schermer M, Willems D, de Vries M (2020). Ethical advice for an intensive care triage protocol in the COVID-19 pandemic: Lessons learned from the Netherlands. Public Health Ethics.

[CR33] Wertheimer A (1983). Jobs, qualifications, and preferences. Ethics.

[CR34] Young I (1990). Justice and the politics of difference.

